# Role of Activating Transcription Factor 4 in Murine Choroidal Neovascularization Model

**DOI:** 10.3390/ijms22168890

**Published:** 2021-08-18

**Authors:** Hiroto Yasuda, Miruto Tanaka, Anri Nishinaka, Shinsuke Nakamura, Masamitsu Shimazawa, Hideaki Hara

**Affiliations:** Department of Biofunctional Evaluation, Gifu Pharmaceutical University, Gifu 501-1196, Japan; yasuda.yakkou@gmail.com (H.Y.); tanakamiruto.yakkou@gmail.com (M.T.); nishinaka.yakkou@gmail.com (A.N.); nakamuras@gifu-pu.ac.jp (S.N.); shimazawa@gifu-pu.ac.jp (M.S.)

**Keywords:** activating transcription factor 4, neovascular age-related macular degeneration, choroidal neovascularization, integrated stress response, ISRIB, vascular endothelial growth factor, autocrine, endothelial cell

## Abstract

Neovascular age-related macular degeneration (nAMD) featuring choroidal neovascularization (CNV) is the principal cause of irreversible blindness in elderly people in the world. Integrated stress response (ISR) is one of the intracellular signals to be adapted to various stress conditions including endoplasmic reticulum (ER) stress. ISR signaling results in the upregulation of activating transcription factor 4 (ATF4), which is a mediator of ISR. Although recent studies have suggested ISR contributes to the progression of some age-related disorders, the effects of ATF4 on the development of CNV remain unclear. Here, we performed a murine model of laser-induced CNV and found that ATF4 was highly expressed in endothelial cells of the blood vessels of the CNV lesion site. Exposure to integrated stress inhibitor (ISRIB) reduced CNV formation, vascular leakage, and the upregulation of vascular endothelial growth factor (VEGF) in retinal pigment epithelium (RPE)-choroid-sclera complex. In human retinal microvascular endothelial cells (HRMECs), ISRIB reduced the level of ATF4 and VEGF induced by an ER stress inducer, thapsigargin, and recombinant human VEGF. Moreover, ISRIB decreased the VEGF-induced cell proliferation and migration of HRMECs. Collectively, our findings showed that pro-angiogenic effects of ATF4 in endothelial cells may be a potentially therapeutic target for patients with nAMD.

## 1. Introduction

Neovascular age-related macular degeneration (nAMD) has been associated with pathological angiogenesis in the form of choroidal neovascularization (CNV) which can progress to a severe reduction of vision [1,2]. Vascular endothelial growth factor (VEGF) is known to be a key mediator for the development of CNV, which are hallmark indicators of nAMD [3,4]. At present, intravitreal injection of anti-VEGF drugs is a commonly used therapy for nAMD, and its use has led to a significant improvement in the prognosis of nAMD [5,6]. While the anti-VEGF therapy has significant therapeutic benefits, some patients respond poorly to anti-VEGF therapy, and they can suffer persistent fluid exudation and vision reduction [7,8]. Thus, a search for other treatment options for nAMD patients is needed to protect nAMD patients from progression to blindness caused by the CNV.

Aging plays a crucial role in the progress of nAMD [9]. The integrated stress response (ISR) is activated during aging, which contributes to age-related disorders [10,11,12]. It was recently shown that the ISR is related to the progression of some eye diseases including glaucoma and proliferative diabetic retinopathy [13,14]. ISR is a common adaptive and evolutionarily conserved intracellular signaling of eukaryotic cells, and it responds to diverse stressful conditions [15]. ISR induces a phosphorylation of eukaryotic initiation Factor2α (eIF2α), and the phosphorylated-eIF2α blocks the eIF2B-mediated GTP-exchange reaction. This reaction results in the suppression of Cap-dependent translation, and the translation of activating transcription factor 4 (ATF4) is facilitated [16,17]. ATF4 functions as a basic leucine zipper (bZIP) transcription factor, and it regulates the expression of some proteins to solve upstream stress conditions [18,19]. Thus, ATF4 is a key mediator of ISR signaling. In addition, ATF4 and cAMP-responsive element binding protein 1 (CREB1) can interact with the ATF4 promoter to enhance a further expression of ATF4 [20]. It was recently found that ATF4 was capable of promoting the angiogenesis of bone, retina, and tumors, mainly by facilitating the expression of VEGF [21,22,23]. Considering that aging is correlated with an upregulation of ISR and the progression of nAMD, it is expected that targeting ISR would suppress the onset or an exacerbation of nAMD.

VEGF has a strong influence on the proliferation and migration of endothelial cells, and it contributes to many types of pathological angiogenesis [24,25]. The level of VEGF expression is higher in the aqueous humor of nAMD patients than in non-nAMD patients [26,27], and it is significantly correlated with the VEGF concentration in the posterior segment of the eye. Karali et al., reported that VEGF signaling promoted the activation of PKR-like ER kinase (PERK)-pathway independently of the endoplasmic reticulum (ER) stress in endothelial cells [28]. Because PERK activates ISR, it is expected that VEGF signaling would trigger ISR and promote angiogenesis by the upregulation of ATF4.

Thus, the purpose of this study was to determine the role played by ATF4 in the formation of CNV and to examine the effects of ATF4 interference in the angiogenesis of retinal endothelial cells. To accomplish this, we used the murine model of laser-induced CNV and determined the effects of the integrated stress response inhibitor (ISRIB), which binds to a regulatory site in eIF2B to downregulate ATF4 [29,30,31,32].

## 2. Results

### 2.1. Level of Expression of ATF4 Is Increased in Choroidal Neovascularization (CNV) Lesions

**Figure 1:**
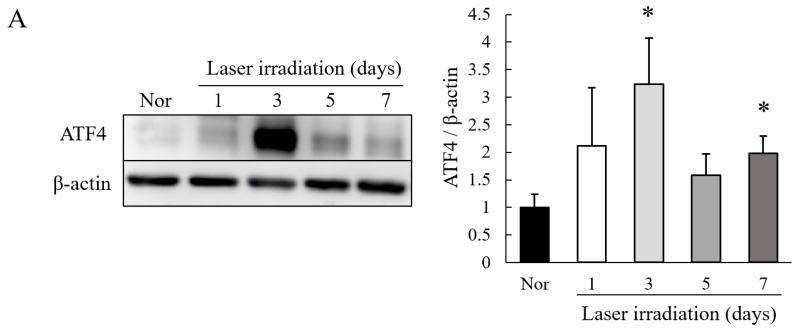

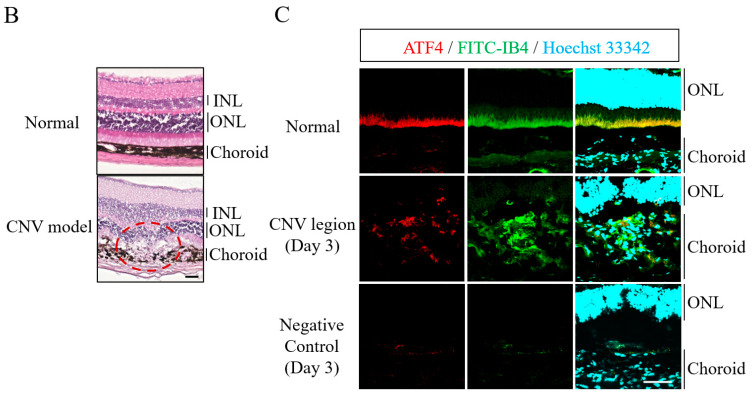
Expression of ATF4 in Laser-induced choroidal neovascularization (CNV). (**A**) Western blot analysis of ATF4 and β-actin in RPE–choroid–sclera complex with laser-induced CNV lesion at 1, 3, 5 and 7 days after the laser irradiation and without CNV treatment (normal, Nor) group. Data are shown as means ± standard error of the measurements (SEMs, *n* = 5). * *p* < 0.05 vs. normal group (Student’s *t*-test). (**B**) Hematoxylin and eosin (H&E) staining of a normal mouse (Normal) and the CNV model mouse at 3 days after the laser irradiation (CNV model). The red dotted line shows the laser irradiation site. (**C**) Immunohistochemistry of ATF4 (red), IB4 (green), and Hoechst 33342 (Blue) at 3 days after the laser irradiation [CNV lesion (Day 3)] and Normal. For the negative control, the cross-section of CNV lesion at 3 days after the laser irradiation was stained with secondary antibody and Hoechst 33342. The scale bars are 50 µm (**B**,**C**). ONL, outer nuclear layer; INL, inner nuclear layer.

To determine the level of expression of ATF4 in the CNV, we used the laser-induced CNV model, which is a common model of nAMD [33,34,35]. After the laser irradiation, the expression of ATF4 was increased in the CNV lesion in the retinal pigment epithelium (RPE)–choroid–sclera complex (Figure 1A). The highest level of upregulation of ATF4 occurred on Day 3. To determine which type of cells express ATF4 in the CNV lesion, we immunohistochemically stained the cross-section of the eye 3 days after the laser irradiation. Our results showed that the expression of ATF4 was colocalized with endothelial cell marker [isolectin B4 (IB4)] at the site of the lesions in the CNV (Figure 1B,C).

### 2.2. ISR Inhibitor, ISRIB, Suppressed CNV Formation

**Figure 2:**
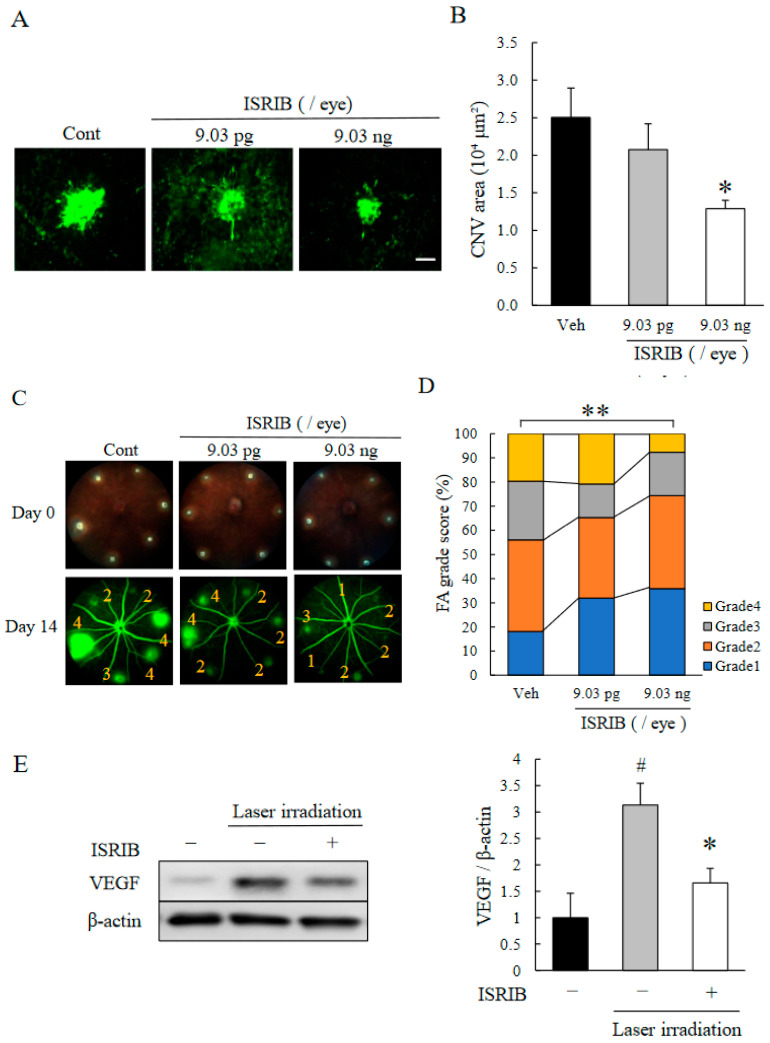
Inhibitory Effect of ISRIB on laser-induced CNV. (**A**–**D**) Just after the CNV induction, vehicle, 9.0 pg/eye (10 nM) or 9.0 ng/eye (10 µM) ISRIB were injected intravitreally. (**A**) Representative photographs and (**B**) quantitative data of their CNV areas in flat mounts. The scale bar is 200 µm. Data are shown as means ± SEMs (*n* = 11–13). * *p* < 0.05 vs. Vehicle (Dunnett’s T3 test). (**C**) Representative images of fundus (day 0) and fluorescein leakage (day 14) in CNV lesions are shown and graded at 14 days after the laser irradiation. (**D**) Quantitative results of the percentage of each grade (grade 1–4) are shown. Data are shown as means ± SEMs (*n* = 11–13). ** *p* < 0.01 vs. Vehicle (Mann–Whitney’s *U*-test). (**E**) Western blot analysis of VEGF and β-actin in RPE–choroid–sclera complex at 3 days after the laser irradiation. Immediately after the laser-irradiation, 9.0 ng/eye (10 µM) ISRIB was injected intravitreally. Data are shown as means ± SEMs (*n* = 4). * *p* < 0.05 vs. Vehicle. ^#^ *p* < 0.01 vs. Control (Student’s *t*-test).

To determine whether the suppression of ATF4 leads to an attenuation of CNV formation, we administrated ISRIB [30,31,32] by intravitreal injection in a laser-induced CNV model. We determined the size of the CNV and the degree of vascular leakage from the new vessels. The injection of ISRIB resulted in a reduction in the formation of CNV (Figure 2A,B) and the fluorescein leakage from the new vessels (Figure 2C,D) in a dose-dependent manner. In addition, an intravitreal injection of 9.0 ng/eye (10 µM, 2µL/eye) of ISRIB reduced the expression of VEGF in the RPE–choroid–sclera complex compared with the vehicle-treated eye (Figure 2E).

### 2.3. ISRIB Suppressed Expression of Pro-Angiogenic Factors Induced by ER Stress

**Figure 3:**
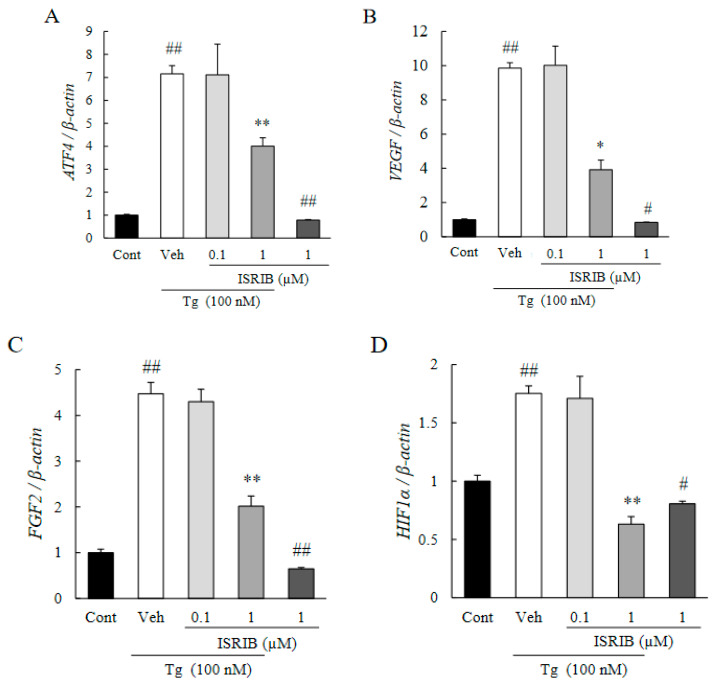
ISRIB attenuates mRNA of ATF4 and pro-angiogenic factor induced by severe ER stress. (**A**–**D**) Level of expression of the mRNA of ATF4 (**A**), VEGF (**B**), FGF2 (**C**), and HIF-1α (**D**) induced by 100 nM of thapsigargin (Tg) for 6 h were determined by qRT-PCR. Thapsigargin was added 1 h after the ISRIB exposure (0.1 or 1 µM). Data are shown as means ± SEMs (*n* = 5 or 6). * *p* <0.05, ** *p* < 0.01 vs. Vehicle (Dunnett’s test), ^#^ *p*< 0.05, ^##^ *p* < 0.01 vs. Control (Welch’s *t*-test).

The pro-angiogenic factors were upregulated by the excessive ER stress in the CNV lesions [36]. In human retinal microvascular endothelial cells (HRMECs), the induction of ER stress by thapsigargin (100 nM; Tg) increased the expression of the mRNAs of ATF4 and pro-angiogenic factors including VEGF, fibroblast growth factor 2 (FGF2), and hypoxic inducible factor-1α (HIF-1α) (Figure 3A–D). On the other hand, exposure of the HRMECs to 1 µM of ISRIB significantly reduced the upregulation induced by thapsigargin. A single treatment of 1 µM of ISRIB also suppressed their expression compared to the control group.

### 2.4. eIF2α-ATF4 Pathway Regulated Autocrine Loop of VEGF in HRMECs

**Figure 4:**
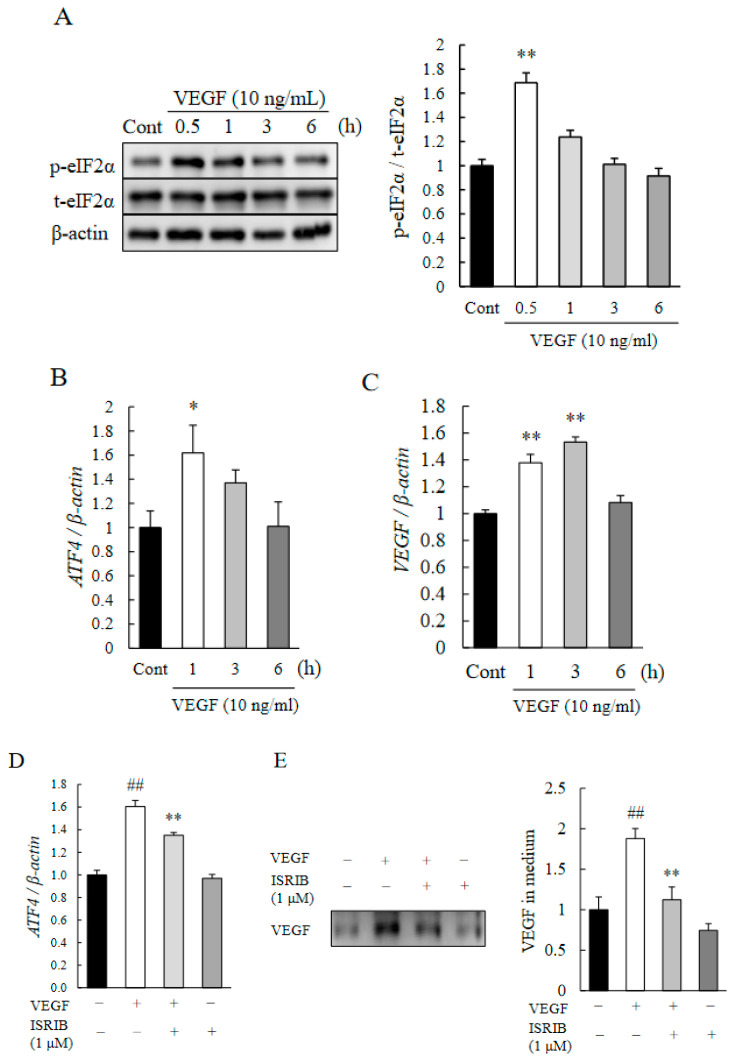
ISRIB attenuates the expression of ATF4 and VEGF induced by rhVEGF. HRMECs were cultured at a density of 4 × 10^4^ cells/mL and incubated for 24 h. (**A**) Phosphorylated level of eIF2α induced by 10 ng/mL rhVEGF was determined by Western blotting. Samples were collected at 0.5, 1, 3, and 6 h after rhVEGF exposure. Data are shown as means ± SEMs (*n* = 5). ** *p* < 0.01 vs. Control (Dunnett’s test). (**B**,**C**) Level of expression of the mRNAs of ATF4 (**B**) and VEGF (**C**) induced by 10 ng/mL rhVEGF were determined by qRT-PCR. Samples were collected at 1, 3, and 6 h after rhVEGF exposure. Data are shown as means ± SEMs (*n* = 6). ** *p* < 0.01, * *p* < 0.05 vs. Control (Student’s *t*-test for B, Dunnett’s test for C). (**D**) Level of Expression of ATF4 induced by 10 ng/mL rhVEGF and the inhibitory effects of ISRIB were determined by qRT-PCR. For this, 1 µM ISRIB was added 1 h before the exposure to rhVEGF. Samples were collected at 1 h after rhVEGF exposure. Data are shown as mean ± SEM (*n* = 5 or 6). ** *p* < 0.01 vs. Vehicle (Dunnett’s test), ^##^ *p* < 0.01 vs. Control (Student’s *t*-test). (**E**) Endogenous VEGF secretion into medium from HRMECs induced by rhVEGF and ISRIB. The level of expression of VEGF was determined by Western-blotting. Data are shown as means ± SEMs (*n* = 6). ** *p* < 0.05 vs. Vehicle (Dunnett’s test), ^##^ *p* < 0.01 vs. Control (Student’s *t*-test).

It has been shown that VEGF activates the PERK pathway in endothelial cells [28], which could initiate angiogenic signaling pathways and autocrine VEGF production. Thus, we investigated the effects of VEGF stimulation on the eIF2α-ATF4 pathway in HRMECs. After exposure to rhVEGF, an upregulation of the phosphorylation of eIF2α and upregulation of ATF4 (Figure 4A,B) were observed. An exposure to rhVEGF also upregulated the endogenous expression of VEGF in HRMECs (Figure 4C). However, exposure to 1 µM of ISRIB reduced the expression of the mRNA of ATF4 induced by rhVEGF (Figure 4D). These findings suggest that ATF4 is essential for the autocrine expression of VEGF in HRMECs. We then analyzed the endogenous expression of VEGF. As shown in Figure 4E, although rhVEGF exposure increased the endogenous VEGF protein secretion into the culture medium, 1 µM of ISRIB exposure significantly reduced the upregulation of endogenous VEGF expression.

### 2.5. ISRIB Inhibited Proliferation and Migration of HRMECs

**Figure 5:**
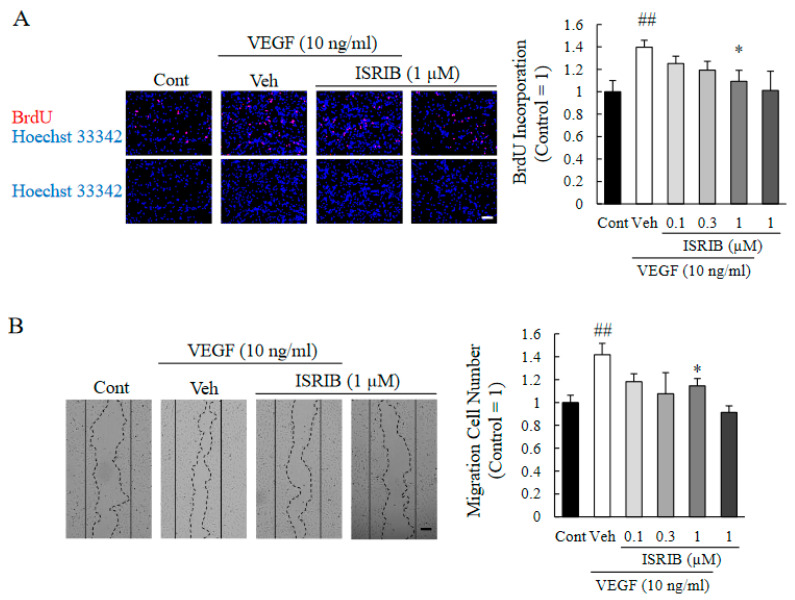
ISRIB attenuates HRMEC proliferation and migration induced by rhVEGF. (**A**) HRMEC proliferation was assessed using the BrdU incorporation assay. Proliferation rates were calculated by the ratio of the number of BrdU-positive cells to total cells. The scale bar is 400 µm. Data are shown as means ± SEMs (*n* = 7). * *p* < 0.05 vs. Vehicle. ^##^ *p* < 0.01 vs. Control (Student’s *t*-test). (**B**) Cell migration was assessed by wound healing assay. Typical images are shown at 24 h after producing the scratch. The number of cells that invaded the wound region was counted as the migration cell number. The scale bar indicates 200 µm. Data are shown as means ± SEMs (*n* = 5). * *p* < 0.05 vs. Vehicle. ^##^ *p* < 0.01 vs. Control (Student’s *t*-test).

To investigate the anti-angiogenic effects of ISRIB on retinal endothelial cells, we performed 5-bromo-2′-deoxyuridine (BrdU) incorporation assays to assess the cell proliferation (Figure 5A), and a wound healing assay to assess the cell migration (Figure 5B). Exposure of HRMECs to rhVEGF increased the cellular proliferation and migration compared to the control group, while ISRIB exposure suppressed the rhVEGF-induced proliferation and migration in a concentration-dependent manner. More specifically, 1 µM of ISRIB significantly suppressed both the rhVEGF-induced proliferation and migration of retinal endothelial cells.

## 3. Discussion

Our results showed that there was an upregulation of ATF4 in the CNV especially in the endothelial cells of the new vessels (Figure 1A–C). In addition, ISRIB, an ISR inhibitor, inhibited the CNV formation, reduced the vascular leakage, and blocked the upregulation of VEGF (Figure 2A–E). It was estimated that the volume of the vitreous cavity in mice was about 5.3 µL [37], and we administrated 2 µL of solution. Considering that ISRIB was diluted and metabolized, it was assumed that the concentration reaching the CNV lesion was about one-tenth of that injected. Because the administration of ISRIB at 10 µM (9.0 ng/eye), but not at 10 nM (9.0 pg/eye), attenuated CNV formation and vascular leakage, it was expected that ISRIB at around 1 µM had inhibitory activity in the murine model of CNV formation. Correspondingly, 1 µM of ISRIB suppressed the ATF4 production induced by thapsigargin (Figure 3A) and rhVEGF (Figure 4D), and the proliferation and migration of HRMECs (Figure 5A,B). In earlier studies, nearly identical concentration of ISRIB reduced the production of ATF4 induced by thapsigargin in ovarian cancer cells and HEK293 cells [38,39]. Our results indicated that ISRIB administration could possibly lead to selective suppression of ATF4 expression in CNV lesions.

In CNV lesions, ER stress, one of upstream ISR, can be induced by several factors including hypoxia, inflammation, and oxidative stresses in the early stage [36]. Hypoxic and ischemic conditions can lead to the upregulation of ATF4 [40,41], and ATF4 enhances the expression of VEGF and some pro-angiogenic cytokines in endothelial cells [42]. Thus, a hyperactivation of ISR can be a cause of pathological angiogenesis. An earlier study showed that genetic deletion of ATF4 reduced the expression of VEGF and resulted in attenuating the degree of angiogenesis in the oxygen-induced-retinopathy (OIR) model that was used to study retinal pathological angiogenesis [22]. Huang et al., showed that ATF4 increased the expression level of monocyte chemoattractant protein-1 (MCP-1) in retinal endothelial cells and enhanced the recruitment of macrophages [43]. VEGF and MCP-1 are also associated with CNV formation and are strongly expressed during angiogenesis in the murine model of laser-induced CNV [44]. Our results showed that ATF4 was expressed in the CNV lesion especially on endothelial cells (Figure 1A). In addition, an intravitreal injection of ISRIB suppressed the expression of VEGF in the RPE-choroid-sclera complex after the laser irradiation (Figure 2E). These findings indicated that the hypoxic condition in CNV lesions can induce an upregulation of ATF4 and may also induce pro-angiogenic cytokines such as VEGF and MCP-1. Thus, hyperactivation of ISR caused by ER stress in CNV lesion can trigger the formation of CNV.

VEGF is a one of the key mediators of CNV formation. In the choroidal endothelial cells, VEGF is produced by the autocrine loop, and it was sufficient to promote angiogenesis [45]. VEGF signaling in endothelial cells activated the ATF6 and PERK pathways via a phospholipase C (PLC)-γ-mammalian target of rapamycin complex 1 (mTORC1)-Akt-dependent signaling [28]. After ATF4 translocated into the nucleus, ATF4 bound to regulatory sites of the VEGFA gene and promoted VEGFA transcription [46]. In retinal endothelial cells, endogenous VEGF expression was increased at 1 to 3 h after exogenous VEGF stimulation, which indicated that a transient upregulation of ATF4 at 1 h may induce the transcription of VEGF (Figure 4B,C). In addition, an exposure to 1 µM of ISRIB suppressed the expression of the mRNA of ATF4 and secretion of VEGF (Figure 4D,E). These results indicated that ATF4 may partly facilitate the expression of VEGF, that is, ATF4 can be a key component of autocrine VEGF signaling. In CNV lesions, RPE cells and bone marrow-derived cells are also known to be sources of VEGF [47,48,49], so to determine their involvements with ATF4 may lead to further understanding on the relationship of ISR on CNV formation. Collectively, ISRIB may attenuate not only the expression of pro-angiogenic factors but also endothelial autocrine VEGF signaling by the eIF2α-ATF4 pathway during CNV formation.

In conclusion, we have shown that ATF4 is associated with choroidal angiogenesis. The results indicate that targeting ISR in endothelial cells might be a potential therapeutic target for treatment of nAMD. However, this study was based on the use of a mouse model of laser-induced CNV, and it is unclear how ATF4 practically functions in the formation of CNV featuring drusen deposits in human. Therefore, additional information is needed to clarify the relationship between ATF4 and human intraocular angiogenesis.

## 4. Materials and Methods

### 4.1. Animals

All investigations were performed in accordance with the Association for Research in Vision and Ophthalmology (ARVO) Statement on the Use of Animals in Ophthalmic and Vision Research. Additionally, the protocols of all animal experiments were approved by the Animal Experimental Committee of the Gifu Pharmaceutical University. In this study, 8-week-old C57BL/6J mice (Japan SLC Ltd., Hamamatsu, Japan) were used for all animal experiments. Animals were housed under a 12-h light/12-h dark cycle at 24 °C ± 2 °C and had free access to standard food and water.

### 4.2. Laser-Induced CNV Model

Eight-week-old mice were anesthetized by an intramuscular injection of a mixture of ketamine (43.8 mg/kg; Daiichi Sankyo Propharma, Tokyo, Japan) and xylazine (2.5 mg/kg; Bayer Healthcare, Tokyo, Japan). The pupils were dilated with 0.5% tropicamide and 0.5% phenylephrine (Santen Pharmaceuticals Co., Ltd., Osaka, Japan). Laser photocoagulation was performed with a 647 nm laser (MC500, NIDEC, Kyoto, Japan), and 100 msec duration and 100 mW power of the laser produced 50 µm spot size on day 0. Six laser spots were made around the optic disc as described in detail [50]. A successful laser photocoagulation was confirmed by the immediate appearance of a bubble, which indicated a rupture of Bruch’s membrane. Thirty laser spots were created per eye for the Western blot analyses. 

On 1, 3, 5, and 7 days after the laser irradiation, mice were euthanized by decapitation. Their eyes were enucleated, and the cornea, lens, and retina were removed. Then, the RPE-choroid-sclera complex was quickly frozen in dry ice and homogenized in cell lysis buffer using a homogenizer (Physcotron; Microtec Co., Ltd., Chiba, Japan).

### 4.3. Intravitreal Injections

The intravitreal injections were performed as described [51] with the following modifications. Immediately after the laser coagulation, 9.0 pg or 9.0 ng of ISRIB (Cayman Chemical, Ann Arbor, MI, USA) in 2 µL [10 nM or 10 µM, dissolved in phosphate-buffered saline (PBS) containing 0.1% dimethyl sulfoxide (DMSO)] was injected into the vitreous cavity with a Hamilton glass syringe (701N; Hamilton Co., Reno, NV, USA) fitted with a 34-G nanopass needle (Terumo, Tokyo, Japan). Then, 5 µL of 0.01% levofloxacin ophthalmic solution (Santen Pharmaceuticals Co., Ltd., Osaka, Japan) was applied topically.

### 4.4. Fluorescein Angiography

Fluorescein angiography (FA) was performed with a MicronIV Retinal Imaging Microscope (Phoenix Research laboratories, Pleasanton, CA, USA). At 14 days after the laser irradiation, mice were anesthetized by an intramuscular injection of a mixture of ketamine and xylazine. The pupils were dilated and topical 0.1% sodium hyaluronate (Santen Pharmaceuticals Co., Ltd., Osaka, Japan) was used to keep the cornea moist. After the tail vein injection with saline was used to confirm successful insertion into the vein, 0.1 mL fluorescein (10 mg/mL; Alcon Japan Ltd., Tokyo, Japan) was injected. The fluorescent images were recorded at 1 and 3 min after the injection of fluorescein and these photographs were defined as early and late phase images. The degree of fluorescein leakage was scored as described in detail [50].

### 4.5. Measurements of CNV Area

After the FA, the mice were perfused with 0.5 mL PBS containing 20 mg/mL of fluorescein-conjugated dextran (MW = 2000 kDa, Sigma-Aldrich, St. Louis, MO, USA) for 5 min. The eyes were then removed and fixed with 4% paraformaldehyde (PFA) for 2 h at room temperature (RT). Then the lens, cornea, and retina were removed, and the RPE-choroid-sclera complex was flat-mounted on glass slide (Matsunami, Osaka, Japan) using Fluoromount (Diagnostic Bio Systems, Pleasanton, CA, USA). The CNV regions were examined and photographed with a fluorescence microscope (BZ-X710; Keyence, Osaka, Japan). The size of the CNV lesions was measured in a masked manner.

### 4.6. Histologic Analysis

The enucleated eyes were fixed in 4% PFA for 24 h at 4 °C. They were then dehydrated in 25% sucrose and stored overnight at 4 °C. They were embedded in optimal cutting temperature compound. The eyes were sectioned at 10 µm thickness with a cryostat (Leica Microsystems, Bensheim, Germany), and the sections were flat mounted on glass slides and washed twice with PBS. The sections were stained with H&E, and the representative images were photographed with a fluorescence microscope. Moreover, for immunofluorescence, the sections were permeabilized with 0.3% Triton-X 100 for 1 h at RT. Then the sections were rinsed in PBS for 5 min and then incubated in PBS containing 10% horse serum (Vector Laboratories, Burlington, VT, USA). After washing with PBS for 5 min, the sections were incubated with ATF4 rabbit monoclonal antibody (1:50, Cell Signaling Technology, Danvers, MA, USA) and fluorescein-labeled Griffonia simplicifolia lectin I (GSL I) IB4 (20 µg/mL: Vector Laboratories, Burlington, VT, USA) overnight at 4 °C.

The slides were then rinsed twice with PBS for 5 min and incubated with Alexa Fluora-546 Donkey anti-rabbit IgG (1:1000, Thermo Fisher Scientific, Waltham, MA, USA) for 1 h at RT. The nuclei were stained with Hoechst 33342 (1:1000, Thermo Fisher Scientific, Waltham, MA, USA) for 10 min at RT. The slides were washed three times with PBS and mounted with Fluoromount. The images were photographed with a FLUOVIEW FV3000 (Olympus, Tokyo, Japan).

### 4.7. Cell Cultures

Primary HRMECs (Cell Systems, Kirkland, WA, USA) were cultured as described in detail [52]. The HRMECs were maintained in complete classic medium supplemented with CultureBoost-R (Cell Systems, Kirkland, WA, USA) and 100 μg/mL of streptomycin (Meiji Seika Pharma Co., Ltd., Tokyo, Japan) and 100 U/mL penicillin (Meiji Seika Pharma Co., Ltd., Tokyo, Japan). Before the cell seeding, the culture dishes and well plates were precoated with an attachment factor (Cell systems, Kirkland, WA, USA). The cells were maintained at 37 °C in a humidified atmosphere with 5% CO_2_. Passage 6 to 10 were used for the experiments.

### 4.8. Western Blots

HRMECs were seeded at 4 × 10^4^ cells/mL and incubated for 24 h at 37 °C with 5% CO_2_. The culture medium was exchanged with a complete medium containing 10% fetal bovine serum (FBS; Biosera, Kansas City, MO, USA) without CultureBoost-R and the cells were incubated for 24 h. The cells were then stimulated with 10 ng/mL recombinant human VEGF (rhVEGF: R&D Systems Minneapolis, MN, USA) and collected at 0.5, 1, 3, and 6 h after the beginning of the rhVEGF exposure.

Tissues or cultured cells were collected in cell lysis buffer composed of a radioimmunoprecipitation buffer containing 0.5% sodium deoxycholate (Wako, Osaka, Japan), 150 mM sodium chloride (Kishida Chemical, Osaka, Japan), 1% Igepal CA-630 (Sigma-Aldrich, St. Louis, MO, USA), 50 mM tris-hydrochloride (Nacalai Tesque, Kyoto, Japan), 0.1% sodium dodecyl sulfate (SDS) (Wako, Osaka, Japan), and protease inhibitor cocktail (Sigma-Aldrich, St. Louis, MO, USA) and phosphatase inhibitor cocktails (Sigma-Aldrich, St. Louis, MO, USA). Then, the samples were centrifuged at 12,000× *g* for 20 min at 4 °C, and the supernatant was collected and used for the following studies. The protein concentration in the extracts was determined using the bicinchoninic acid protein assay kit (Pierce Biotechnology, Rockford, IL, USA) with bovine serum albumin as the standard.

The protein sample and sample buffer (Wako, Osaka, Japan) were admixed at a ratio of 3:1 and boiled for 5 min. The samples were separated by gradient of 5–20% SDS-PAGE (Wako, Osaka, Japan). The proteins were transferred to polyvinylidene difluoride membranes (Immobilon-P; Millipore, Bedford, MA, USA), and after the membranes were washed with tris-buffered saline (TBS) containing 0.05% Tween20 (Bio-Rad, Hercules, CA, USA), they were incubated with Blocking One-P (Nacalai Tesque, Kyoto, Japan) for 30 min at RT. Then, the membranes were incubated with primary antibodies overnight at 4 °C. The following primary antibodies were used: ATF4 rabbit monoclonal antibody (1:1000, Cell Signaling Technology, Danvers, MA, USA), β-actin mouse monoclonal antibody (1:2000, Sigma-Aldrich, St. Louis, MO, USA), VEGF mouse monoclonal antibody (1:200, Santa Cruz biotechnology, CA, USA; in vivo), VEGF rabbit polyclonal antibody (1:1000, Merck Millipore, Burlington, VT, USA; in vitro), phospho-eIF2α rabbit polyclonal antibody (1:1000, Cell Signaling Technology, Danvers, MA, USA), and eIF2α rabbit polyclonal antibody (1:1000, Cell Signaling Technology, Danvers, MA, USA).

After the incubation with the primary antibodies, the membranes were washed in TBS containing 0.05% Tween-20. Then, the membranes were incubated with horseradish peroxidase (HRP)-conjugated goat anti-rabbit IgG (1:2000, Thermo Fisher Scientific, Waltham, MA, USA) and HRP-conjugated goat anti-mouse IgG (1:2000, Thermo Fisher Scientific, Waltham, MA, USA) overnight at 4 °C. The immunoreactive bands were made visible by ImmunoStar LD (Wako, Osaka, Japan). The intensities of the bands were measured by an imaging analyzer (Amersham Imager 680; GE Healthcare, Chicago, IL, USA).

### 4.9. Measurements of Endogenous VEGF Secreted from HRMECs

HRMECs were seeded at 4 × 10^4^ cells/mL and incubated for 24 h at 37 °C with 5% CO_2_. The culture medium was exchanged with the complete medium containing 10% FBS without CultureBoost-R, and the cells were incubated for 24 h. Then, 1 µM ISRIB was added to the culture medium and incubated for 1 h. Then, 10 ng/mL of rhVEGF was added to each well and the cells were incubated for 24 h. Then, the medium was removed, and cells were rinsed with PBS twice to remove the exogenous VEGF. After another 24 h of incubation in flesh medium containing 1% FBS, the medium was collected and applied to centrifugal filter devices (Amicon Ultra—0.5; 3.0 kDa MW cut off; Merck Millipore, Burlington, VT, USA). The medium was centrifuged at 14,000× *g* for 30 min at 4 °C. The supernatant was collected, and the endogenous VEGF expression was determined by Western blotting.

### 4.10. RNA Isolation and qRT-PCR

HRMECs were seeded at 4 × 10^4^ cells/mL and incubated for 24 h at 37 °C with 5% CO_2_. The culture medium was exchanged with a complete medium containing 10% FBS without CultureBoost-R, and the cells were incubated for 24 h. Then, 1 µM ISRIB was added to the medium and incubated for 1 h. rhVEGF (10 ng/mL) was added to each well and the cells were incubated for 1 h. Then, 100nM of thapsigargin (Wako, Osaka, Japan) was added to each well and the cells were incubated for 6 h. Cells were collected and isolated as previously described [52].

Total RNAs were isolated from cultured cells using NucleoSpin RNA kit (Takara, Shiga, Japan) following the manufacturer’s protocol. Then, the RNA concentration in the RNA extract from cells was determined using NanoVue Plus (GE Healthcare Japan, Tokyo, Japan). The cDNA was synthesized using PrimeScript RT reagent kit (Takara, Shiga, Japan). The quantification of the RNA levels was done with TB Green Premix Ex Taq II (Takara, Shiga, Japan) following the manufacturer’s protocol. The thermal cycling reactions was performed as previously described [52]. The following PCR primers were used: ATF4 forward, AGGAGTTCGCCTTGGATGCCCTG; reverse, AGTGATATCCACTTCACTGCCCAG; VEGF forward, TCTACCTCCACCATGCCAAGT; reverse, GATGATTCTGCCCTCCTCCTT; FGF2 forward, CTAACCGTTACCTGGCTATG; reverse, TTATACTGCCCAGTTCGTTT; HIF-1α forward, CTCAAAGTCGGACAGCCTCA; reverse, CCCTGCAGTAGGTTTCTGCT; and β-actin forward, TCAAGATCATTGCTCCTCCTG; reverse, CTGCTTGCTGATCCACATCTG.

### 4.11. Cell Proliferation Assay

HRMECs were seeded at 3 × 10^4^ cells/mL in 96-well plates and incubated for 24 h at 37 °C with 5% CO_2_. The medium was exchanged with complete classic medium containing 10% FBS without CultureBoost-R, and the cells were incubated for 24 h. Then, 0.1, 0.3, or 1 µM of ISRIB were added into each well and the cells were incubated for 1 h. Then, rhVEGF was then added to each well, and after 36 h of incubation, the cells were incubated with 10 µM of BrdU (Sigma-Aldrich, St. Louis, MO, USA) for another 12 h.

After removing the medium, the cells were fixed in 4% PFA for 15 min at RT. Then, the cells were washed twice with PBS and treated with 2 M HCl for 15 min at RT. After washing with PBS, cells were treated with PBS containing 50 mM glycine (Wako, Osaka, Japan) for 30 min. After blocking, the cells were incubated with PBS containing 3% goat serum (Vector Laboratories, Burlington, VT, USA) for 30 min. After washing with PBS, the cells were incubated with rat anti-BrdU (1:250, Abcam, Cambridge, MA, USA) overnight at 4 °C.

Subsequently, the samples were washed with PBS and incubated with Alexa Fluora-546 Goat anti-rat IgG (1:1000, Thermo Fisher Scientific, Waltham, MA, USA) for 1 h at RT. The nuclei were stained with Hoechst 33342 (1:1000) for 10 min at RT. After washing with PBS three times, the cells were photographed with a fluorescence microscope. The ratio of the BrdU-positive cells in the photographs was calculated.

### 4.12. Wound-Healing Assay

A wound-healing assay was performed as described [53] with the following modifications. The HRMECs were seeded at 1 × 10^5^ cells/mL and incubated for 24 h at 37 °C with 5% CO_2_. The medium was exchanged with a complete classic medium containing 1% FBS without CultureBoost-R, and the cells were incubated for 6 h. The HRMECs monolayers were scratched with a P1000 pipette tip. The cells were washed, and 0.1, 0.3, or 1 μM ISRIB and 10 ng/mL rhVEGF were added to each well. The cells were incubated for 24 h in complete classic medium containing 1% FBS without CultureBoost-R. The images were photographed with a fluorescence microscope. The migration ratio was calculated by counting the number of cells that invaded the scratched region. Three independent fields were assessed in each well.

### 4.13. Statistical Analyses

All values are expressed as the means ± standard error of the means (SEMs). The significance of the differences was determined by the Student’s *t*-tests, Welch’s *t*-tests, Dunnett’s tests, Dunnett’s T3 tests, or Mann-Whitney’s *U* tests. *p* values < 0.05 were taken to be statistically significant. The statistical analyses were performed with the SPSS Statistics software (IBM Corporation, Armonk, NY, USA).

## Data Availability

Data is contained within the article.
